# A large EEG database with users’ profile information for motor imagery brain-computer interface research

**DOI:** 10.1038/s41597-023-02445-z

**Published:** 2023-09-05

**Authors:** Pauline Dreyer, Aline Roc, Léa Pillette, Sébastien Rimbert, Fabien Lotte

**Affiliations:** 1 Centre Inria de l’université de Bordeaux, Talence, 33405 France; 2 LaBRI (Univ. Bordeaux/CNRS/Bordeaux INP), Talence, France; 3 Inria de l’Université de Rennes, CNRS, IRISA, Rennes, 35042 France

**Keywords:** Data processing, Data acquisition, Scientific data

## Abstract

We present and share a large database containing electroencephalographic signals from 87 human participants, collected during a single day of brain-computer interface (BCI) experiments, organized into 3 datasets (A, B, and C) that were all recorded using the same protocol: right and left hand motor imagery (MI). Each session contains 240 trials (120 per class), which represents more than 20,800 trials, or approximately 70 hours of recording time. It includes the performance of the associated BCI users, detailed information about the demographics, personality profile as well as some cognitive traits and the experimental instructions and codes (executed in the open-source platform OpenViBE). Such database could prove useful for various studies, including but not limited to: (1) studying the relationships between BCI users’ profiles and their BCI performances, (2) studying how EEG signals properties varies for different users’ profiles and MI tasks, (3) using the large number of participants to design cross-user BCI machine learning algorithms or (4) incorporating users’ profile information into the design of EEG signal classification algorithms.

## Background & Summary

Motor Imagery-based Brain-Computer Interfaces (MI-BCIs) enable their users to interact with digital technologies, such as computer programs or prosthesis, by performing motor imagery tasks only, e.g., by imagining hand movements. This is possible thanks to their brain activity being recorded (usually by electroencephalography; EEG) and processed in real-time by the BCI^1^. MI-BCIs are very promising neurotechnologies for many applications^1^, including rehabilitation and/or as assistive technologies for motor-impaired users^2^ or as a new interaction method for healthy users^3^. Unfortunately, they are currently not reliable enough, with 10–30% of naive users being unable to control MI-BCIs, a phenomenon sometimes called BCI illiteracy/deficiency^4^. Moreover, for many users able to control MI-BCI, the achieved MI task recognition accuracy can still be rather low^5^. Making BCI more effective and usable requires not only improvements on the machine learning side (e.g., by improving brain signal analysis algorithms^6^) but also on the user learning side^7^. Controlling an MI-BCI is indeed a skill that must be learned^8^ and requires practice^9^. Thus, in order to improve user training and thereby the reliability of MI BCIs, it is required to understand the cognitive and neurophysiological processes that underlie this ability to encode mental commands efficiently through the performance of MI, i.e. to produce stable and distinct EEG patterns. The study of MI-BCI user training is crucial to better apprehend the extent to which MI-BCI users can learn when and how to modify their MI strategy, and thus their brain patterns, so that their mental commands are better recognized by the system.

The goal of the BrainConquest project (ERC Starting Grant project, 2017–2022) is precisely to conducted research works that aimed at addressing this problem, i.e., to understand, model and optimize user’s training in order to provide more efficient MI-BCIs^10–12^. As expected, it generated several datasets available to be shared as open data. First, with the *Dataset A* generated by the study of Pillette *et al*.^10^, we investigated the impact of experimenters’ and users’ gender on MI-BCI user training outcomes, i.e., users performance and experience. Indeed, experimenters have a fundamental role, for example they keep users motivated. While providing emotional and social feedback, they introduce users to BCI with clear instructions. Secondly, with the *Dataset B* generated by the study Benaroch *et al*.^11^, we examined the relationship between users’ online performance (i.e., classification accuracy) and the characteristics of the chosen user-specific Most Discriminant Frequency Band (MDFB), by using computational models. Indeed, understanding the processes underlying user-training by modeling it computationally is a key to improve MI-BCI training protocols and adapt them to each user profile. Finally, *Dataset C* contains 6 additional participants (see section Participants) who completed one of the two experiments described above.

Also, the wide variation observed in BCI performance between users remains a critical topic in the field of BCI that has not yet been fully understood and addressed^13,14^. The inclusion of detailed profile data in a large public database is unfortunately rare in the field. Therefore in both these experiments we collected via 6 questionnaires user’s demographic, personality profile as well as some cognitive traits (see subsection Questionnaires). This added value, together with the relatively large number of users can, among others, help the community to: (1) study the relationships between BCI users’ profiles and their BCI performances, (2) study how EEG signal properties vary for different users’ profiles and MI tasks, (3) use the large number of participants to design cross-user BCI machine learning algorithms or (4) incorporate users’ profile information into the design of EEG signal classification algorithms. The database preparation described in this paper was part of a Data Management Plan of the European Research Council Starting Grant Project BrainConquest to make the project data Findable, Accessible, Interoperable and Reusable.

There are a few public EEG-BCI databases about motor BCIs, mostly on motor-imagery and/or sensori-motor BCI and several of these databases include a substantial number of subjects, e.g., 52, 54, 55, 62 and 109 for^15–19^. Among these databases, most do not provide information on the users’ profile. Databases from^16,19^ contain some information on the users’ demographics as well as on their subjective states during BCI use, but no information on their personality or cognitive traits. Only the database from^15^ contains such information, for a database with 52 subjects. Therefore, to the best of our knowledge, our extensive database of EEG and user demographic, personality profiles as well as their cognitive traits that we share and describe in this manuscript, would be the largest BCI database (in terms of number of participants) that includes both EEG data and user profile information. Indeed it contains electroencephalographic (EEG) signals from 87 participants, with more than 20,800 trials in total which represent about 70 hours of recording. It also contains the users’ online performances in the two BCI experiments, a large number of responses (more than 6200 items) included in the 6 questionnaires related to demographic information, spatial abilities, pre- and post-experiment user state, learning style, personality profile and cognitive traits. Moreover, the personality traits we measured are more detailed in our database than in^15^ (16 personality factors for our database versus 5 big personality traits from that of^15^), and our database contains different cognitive traits. We also provide information on the study design and instructions, methods, and codes used to conduct the studies (scenarios and scripts used to run the experiments with the free and open-source BCI platform OpenViBE^20^).

The possibility of making a new publicly available database with information on the cognitive traits of participants is a valuable asset. Indeed, providing researchers with a larger and more diverse dataset could allow the community to expand their research to design new machine learning algorithms or identify new factors associated with BCI performance and learning. Thus, we hope that our database can contribute to the advancement of EEG and BCI research by improving the accuracy and robustness of BCI systems.

## Methods

### Participants

Eighty-seven (87) participants completed one of the two BCI experiments based on the same protocol. They are organized as follows:

- 60 participants (*Dataset A*; 29 women; age 19–59, M = 29, SD = 9.32) performed the study on the impact of experimenters’ and users’ gender^10^.
- 21 participants (*Dataset B*; 8 women; age 19–37, M = 29, SD = 9.318) performed the study on the MDFB study^11^.
- 6 additional participants (*Dataset C*; 4 women; age 20–26, M = 22; SD = 2.34) performed one of the two experiments after the corresponding studies were published (these participants were thus not included in the previously published analyses). Participants C83 and C85 performed the study of Pillette *et al*.^10^ later and the 4 remaining participants performed the study of Benaroch *et al*.^11^ later on.

Recruitment was limited to volunteer participants aged 18–60 years old, with no history of neurological or psychiatric disorders, normal vision (or corrected) and naive MI-BCI user, i.e. using a MI-BCI system for the first time.

Eight different experimenters conducted the experiments. In *Dataset A*, the design of the research question implied a particular balance in the gender of the participants and experimenters. As a results, 3 women and 3 men experimenters each ran the experiment with 5 women and 5 men participants. There was no authority relationship between participants and experimenters as they did not know each other. In the *Dataset B and C*, two other experimenters (2 women) conducted the experiments. The studies were conducted according to the relevant ethical research guidelines set out in the Declaration of Helsinki^21^. Before participating in each study, both participants and experimenters gave informed consent. Participants thus signed an informed consent text in which they agreed both to participate in the studies and to share their EEG and experimental data. In the consent form each participant was asked whether they agreed to have their data used anonymously for publication in journals and conferences. Indeed they were informed that the database will be fully anonymized and all precautions would be taken to protect the privacy of all participants before the corresponding data can be publicly released and shared to the whole research community, in conformity with applicable ethical and legal requirements. We explained that to ensure replicability/reproducibility and to enable others to improve on our work, sharing the collected data on open data platforms was also an objective and outcome of our research projects, which are part of the ERC Open Research Data Pilot program. It was also clarified that they were completely free to end their participation in the experiment at any time without providing any reasons. The studies have been approved and reviewed by Inria’s ethics committee, the COERLE (Approval number: 2018-13).

### EEG data acquisition

**Fig. 1:**
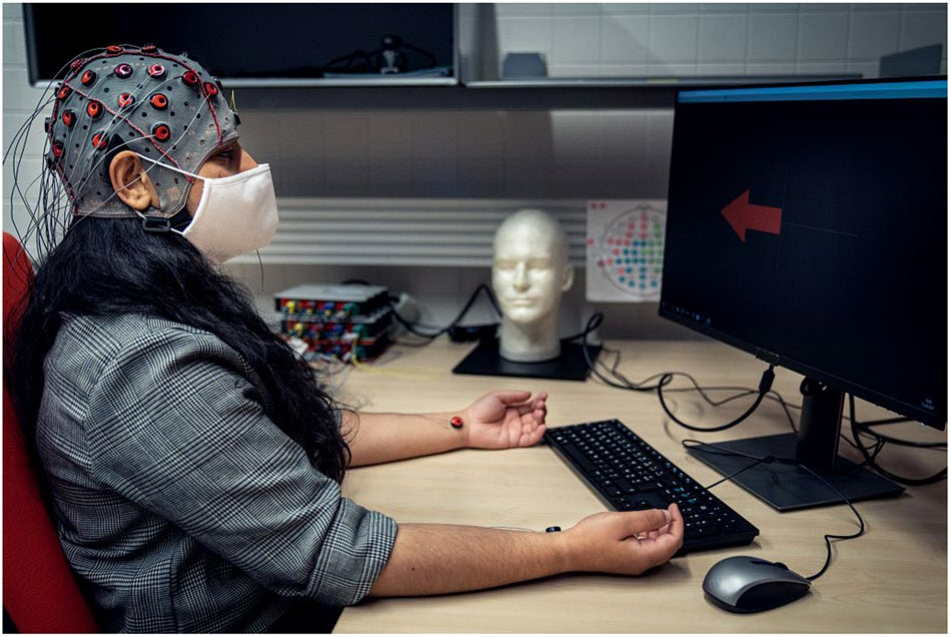
Setup of the experiment, with a participant in front of the computer. The person has given consent for the image to be published ©Inria/Photo B. Fourrier.

Participants sat comfortably in a chair in front of a computer screen see Fig. 1.

**Fig. 2:**
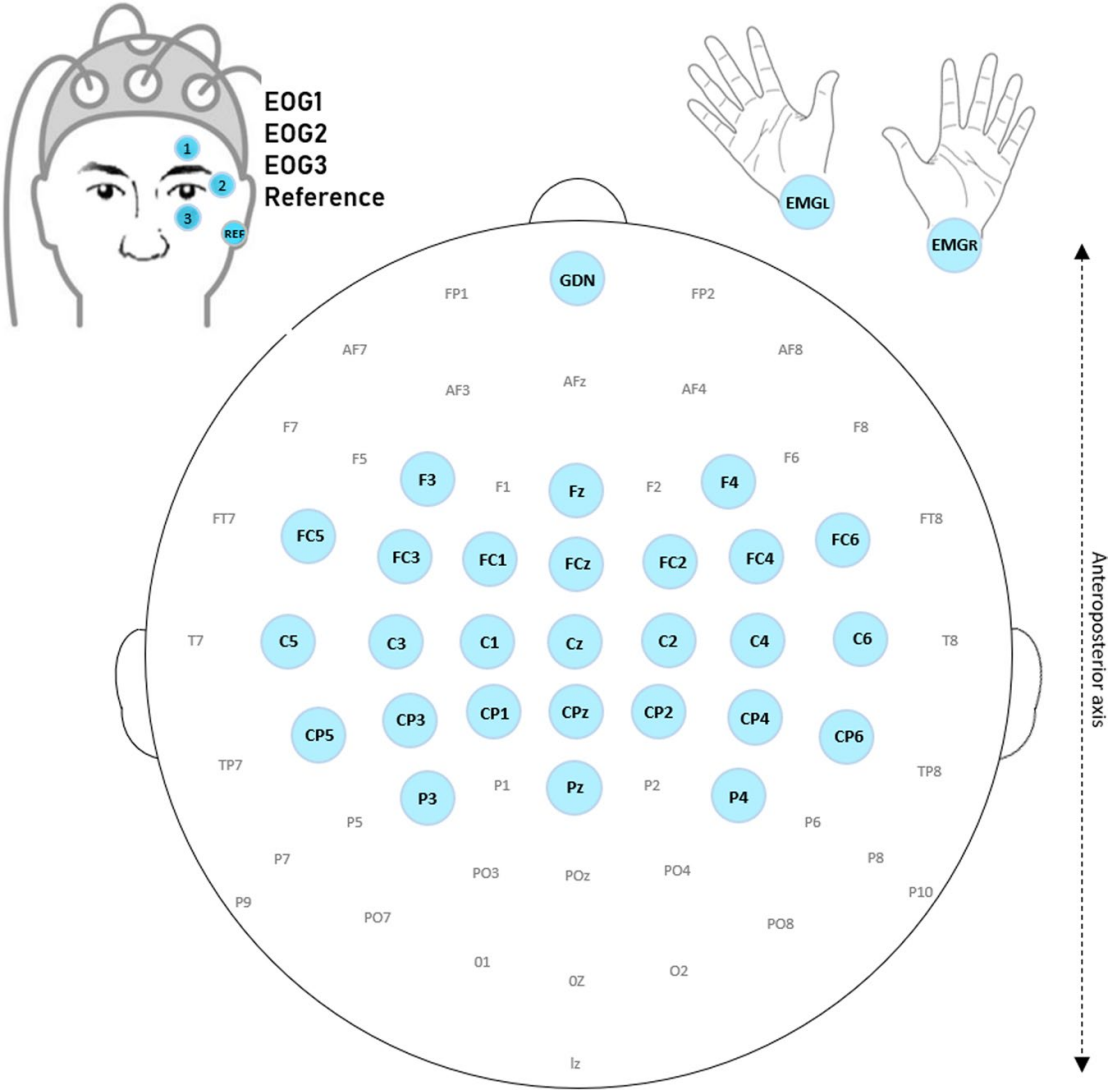
EEG cap used with the location of the active electrodes represented in blue. The reference electrode is on the left earlobe, the ground electrode is placed in FPz position.

EEG data was acquired using 27 active scalp electrodes (i.e., Fz, FCz, Cz, CPz, Pz, C1, C3, C5, C2, C4, C6, F4, FC2, FC4, FC6, CP2, CP4, CP6, P4, F3, FC1, FC3, FC5, CP1, CP3, CP5, P3, 10–20 system), referenced to the left earlobe, the ground electrode is placed in FPz position. Figure 2 represents the 32 active electrodes as well as the reference and the ground electrodes used for the experiments.

The electromyographic (EMG) signals of both hands were recorded using two active electrodes located 2.5 cm below the skinfold on each wrist. The electrooculography (EOG) signals of one eye was recorded using three active electrodes. Two of them were located below and above the eye and one was located on the side. For Dataset A the EOG signals of the right eye were recorded, and then due to a change in the layout of the experimental room, in Dataset B the EOG signals of the left eye were recorded. Physiological signals were measured using two g.USBAmp amplifiers (g.tec, Austria), sampled at 512 Hz, and processed online using the open-source BCI platform OpenViBE 2.1.0 (Dataset A) or OpenViBE 2.2.0 (Dataset B)^20^. In the Dataset C Participants C83 and C85 were collected with OpenViBE 2.1.0 and the remaining 4 participants with OpenViBE 2.2.0. This platform enables to design, study and use BCIs in real-time. The recording room was dimly-lit. The raw signals were recorded without any hardware filters (see all information about the amplifier used https://www.gtec.at/product/gusbamp-research/). Note that with the g.USBamp, impedance check can only be performed with passive electrodes. It is not possible with active electrodes as used in this experiment. Thus, the impedance of the electrodes could not be measured for these two experiments. However, the EEG signals were visually checked by the experimenters, and regularly re-checked, to ensure a good signal quality throughout the experiment.

### Protocol

**Fig. 3:**
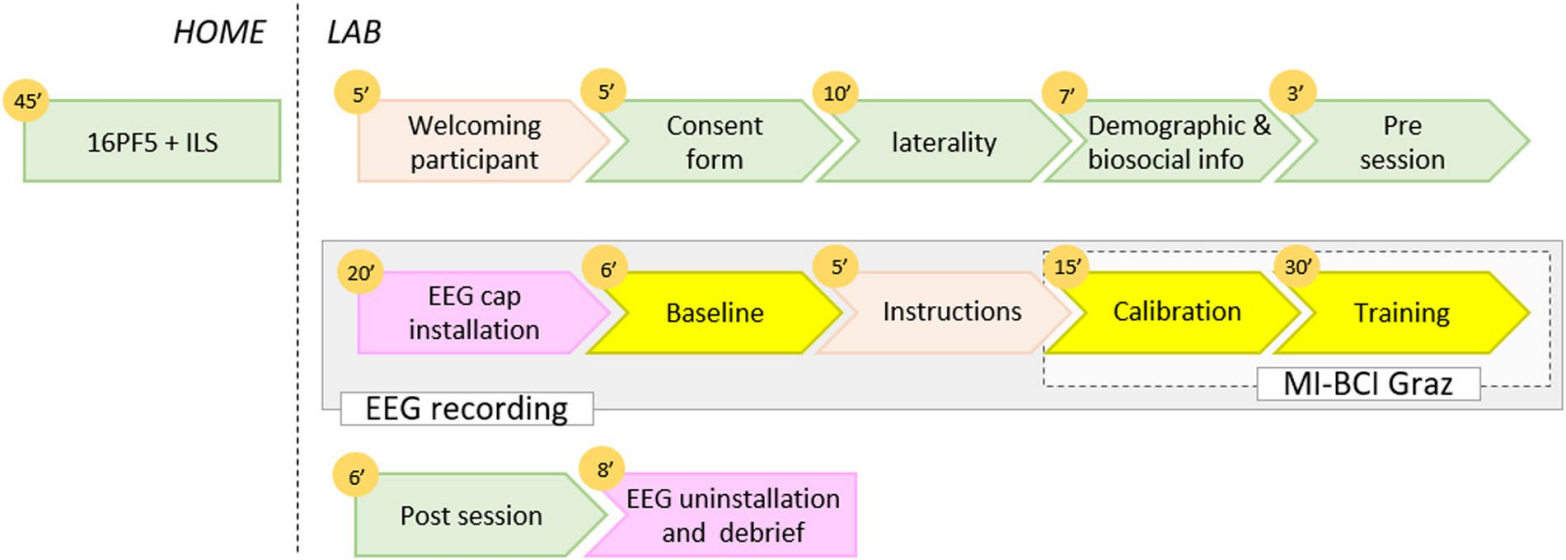
Experimental protocol of the two experiments. The orange circles correspond to the minutes that each part takes. In green rectangles are the questionnaires, in pastel orange rectangles are the experimenter’s instructions, in pink rectangles are the technical processes with the cap, in yellow is the EEG recordings.

Information relative to participants’ demographic, personality profile as well as some cognitive traits were assessed with online questionnaires as a follow-up to existing work on predictors of BCI performance^14^. A couple of days before the BCI session, participants were asked to complete at home two of the six questionnaires collected in the experiment: the 5th edition of the 16 Personality Factors (16PF5)^22^ and the Index of learning style (ILS)^23^. The 6 questionnaires are presented in the questionnaires subsection below. The general experiment workflow described as follow is illustrated in Fig. 3.

Each participant attended one single session that lasted around 2 hours. At the beginning of the session, all participants completed the consent form (in which they all agreed to both participate to the experiment and share their data), followed by 3 questionnaires (see questionnaires section) (around 20 min): a mental rotation test on paper^24^, then a demographic information questionnaire on the computer by and finally a pre-experiment questionnaire on users’ states (the NeXT questionnaire^25^), their consumption of stimulants, and their sleep habits, still on the computer.

This was followed by the installation of the EEG cap (around 20 min). Once the installation done, the experimenter ensured good experimental conditions (e.g., no outside noise, phones turned off…) and the optimal functioning of the equipment (e.g., electrodes, amplifier…). The experimenter showed the participant the different signals appearing on the screen and explained the importance of not generating any muscular activity during the MI task. Then, two EEG baselines (resting state) were recorded with successively opened and closed-eyes (3 min for each condition). During this task participants were instructed to relax and not to move. All the instructions were written down and read orally by the experimenter to the participant so that all the participants started with the same standardized information (cf. the instructions.csv file).

After the baselines and before starting the actual BCI part of the experiment, instructions were provided regarding the course of the session and the mental tasks to perform, i.e., imagine right or left hand movements. We respected the following recommendations: encourage users to perform kinesthetic imagination^26^ and leave them free to choose their mental imagery strategy^27^. Then, the nature of the feedback was explained. The latter was continuously provided, under the form a blue bar varying in length. The length of this blue bar varied according to the BCI classifier output during online BCI use, and reflected the BCI confidence in the recognized MI task (It was thus a real feedback. A sham feedback was used during the calibration runs, before the online BCI use). Participants were instructed to try to find the best strategy so that the system would show the longest possible feedback bar. Only positive feedback was provided, i.e., the feedback was provided only when the recognized task matched the instructed task. Participants had a demonstration of a BCI run to familiarize themselves with the interface (notably the cues and the feedback that appeared on the screen). The participants could ask questions at any point during these explanations. As they would have been in any standard BCI experiment, the experimenters were free to interact with the participants before, during and after the experiment.

**Fig. 4:**
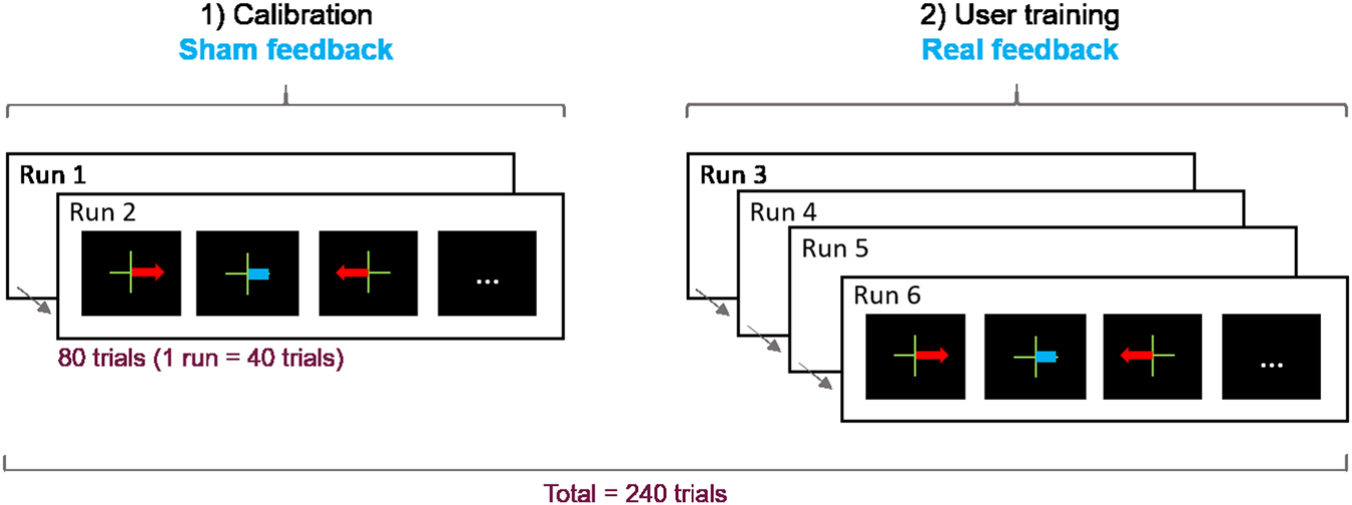
The BCI session included 6 runs divided into two steps: (1) data acquisition to train the system (2 runs, 80 trials in total with sham feedback) and (2) user training (4 runs, 160 trials in total). The classifier was trained after Run 2, on the data collected during the two first runs. Then the user training began with real online feedback.

The demonstration was followed by the MI-BCI session itself, which lasted around 60 min, including breaks between the runs. Participants had to complete six runs (7-minutes each) during which they had to learn to perform the two MI-BCI tasks (imagine right or left hand movements). The training protocol used for this experiment was the “Graz protocol”^28^, divided into two steps: (1) data acquisition, to train the system and (2) user-training, with real-time BCI feedback, see Fig. 4.

The first two runs were used as calibration to provide the system with examples of EEG patterns associated with each of the MI tasks. As the classifier was not yet trained, it could not provide consistent feedback for these two runs. Therefore, equivalent sham feedback was provided for the two calibration runs to limit bias and EEG changes due to differences in visual processing between runs. Similar to the real feedback, the sham feedback was a blue bar of varying length appearing on the side associated with the task that the participant performed (e.g. on the left of the center cue for left hand motor imagery). However, the bar length was random for these two runs of data acquisition (based on previously recorded BCI training data from a different participant).

#### Run procedure

**Fig. 5:**
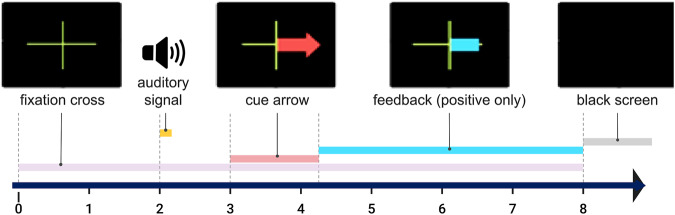
Process of one trial with the different events occurring in time.

During each run, participants performed 40 trials (20 per MI-task, presented in a random order) each trial lasting 8 s. First a green cross appeared (t = 0 s) on the screen, then an acoustic signal (t = 2 s) announced the appearance of a red arrow (t = 3 s). The arrow pointed towards the task to be performed. (e.g., towards the left for left hand MI) and remained displayed for 1.25 s. From t = 4.25 s, the visual feedback was continuously provided (blue bar varying in length). The feedback lasted for 3.75 s and was updated at 16 Hz, using a 1 s sliding window. Positive feedback only was displayed. Then the screen turned black again after 8 seconds until the next trial begin, starting randomly between 1.5 to 3.5 seconds later. (see Fig. 5).

At the end of the session, participants filled-in the post-experiment NeXT questionnaire^25^ (around 5 min). Then, the cap was removed and participants were debriefed (around 8 min).

### BCI performance metrics and signal processing

The BCI design and EEG signal processing correspond to a rather standard approach, that has been used in numerous previous experiments by various laboratories^5,29,30^. Participant-specific spectral and spatial filters are used to classify the Left vs. Right MI tasks from EEG data. To do so, we used the now standard algorithms, in particular the Common Spatial Pattern (CSP) spatial filter algorithm^31^ as well the most discriminant frequency band selection algorithm proposed by Blankertz *et al*.^29^ (Algorithm 1 in that paper).

More precisely, we first identified a participant-specific discriminant frequency band from the EEG signals recorded during the calibration runs, using either the heuristic algorithm proposed by Blankert *et al*.^29^ for *Dataset A* or a variant of it proposed by Benaroch *et al*. (Algorithm 2 in that paper)^11^ for *Dataset B and C*. The heuristic algorithm selects the frequency band whose power in the sensorimotor channels maximally correlates with the class labels. Specifically, we followed the recommendation outlined in^29^ and used channels C3 and C4 as our sensorimotor channels after applying a Laplacian filter for spatial filtering. The algorithm successfully selected a discriminant frequency band in the range of 5 Hz to 35 Hz with 0.5 Hz large bins. Once this discriminant frequency band was automatically identified, we proceeded to filter the EEG signals within this band using a fifth-order Butterworth filter. For the variant algorithm, we added constraints^11^ to the algorithm by imposing a Most Discriminant Frequency Band (MDFB) width greater than 3.5 Hz and a MDFB mean below 16 Hz, as such values were found to correlate with higher classification accuracies^11^. See pages 8 and 9 of the article by Benaroch *et al*.^11^ for the detailed procedure.

Then, still as recommended in^29^, we used the Common Spatial Pattern (CSP) algorithm^31^, to optimize 3 pairs of spatial filters. This optimisation was performed using data from the two calibration runs. Such spatially filtered EEG signals should thus have a band power which is maximally different between the two MI conditions. The band power of these filtered signals was then determined by squaring the EEG signals, averaging them over a 1 second sliding epoch (with a 1/16 second gap between consecutive epochs), and applying a logarithmic transformation to the results.

This yielded to 6 different features per epoch, which were used as input to a Linear Discriminant Analysis (LDA) classifier^6^. It is important to note that the LDA classifier was calibrated using the data from the two calibration runs. These filters and classifier were then implemented on the subsequent runs to provide online feedback. Each participant’s file in the database^32^ (see Data Records section) contains information about the frequency band selected for the experiments.

#### Performances

The metric used for quantifying BCI performances is the online Trial-wise Accuracy (TAcc), i.e. the default performance metric provided online in the MI-BCI scenarios of OpenViBE. Only experimenters were seeing online this metric. It is reported in the “performances.xml” file, where “OpenVibe Perf” contains details of the results for the 4 runs. As the name indicates, TAcc measures the number of trials that were accurately classified. The TAcc for a run was determined by assigning a boolean value - either correct or incorrect classification - to each trial. As an example, a 80% TAcc in a run means that 32 trials were correctly classified out of 40. To determine whether a trial was correctly classified, the classifier (here a Linear Discriminant Analysis - LDA) outputs are used, i.e. the distance to the separating hyperplane, based on^33^ (using one separating hyperplane per class), for all 1s-long epochs, with 15/16 s overlap between consecutive epochs, of the trial. The trial classification outcome was computed by first summing the (signed) LDA outputs (normalization of LDA distances for the two classes labelled left and right: $\frac{left-right}{right+left}$) over all epochs during the trial feedback period (i.e., from t = 4.25 s to t = 8 s of that trial). The trial was considered as correctly classified, if this sum sign matched the required trial label, i.e., negative for left hand MI and positive for right hand MI, then otherwise it was not. The TAcc for each run was calculated as the percentage of trials that were accurately classified using this methodology. It should be noted that this metric uses the LDA output (distance) instead of a discrete classification output for each epoch. Thus, the TAcc metrics also reflects the feedback bar length, which participants saw, as it is proportional to the classifier output. Our participants were instructed to train in order to obtain not only a correct classification result, but also, as mentioned above, a feedback bar for as long as possible, so the TAcc metrics take both aspects into account.

### Questionnaires

As the personality and cognitive profile of participants and experimenters can influence BCI performance and experimenter bias^34^, we assessed the personality and some cognitive traits of both participants and experimenters in both experiments using the 5th edition of the 16 Personality Factors (16PF5)^22^. Previous results suggest that there is a correlation between scores in some dimensions of the 16PF5 psychometric questionnaire and the control of a BCIs,^14^. Participants also completed pre- and post-experiment questionnaires (before and after the sessions), which assessed participants’ states and user experience (UX)^25^).

The answers to the questionnaires filled-in by participants and/or the scores computed from the answers are fully reported in the “Performances.xlsx” file. The questionnaires described in this section were all completed in French by the participants. A translated (English) version of the questionnaires is provided in the “TranslationQuestionnaires” file. All questionnaires used in the two experiments are described below. The identification of the columns in the “Performance.xlsx” file is given at the beginning of each question.

#### Demographic information

Answers to the demographic questionnaire are provided as numbers, using the code below, where each answer is associated with a numerical code. As mentioned above each item corresponds to the name of a column in the “Performance.xlsx” file.

- SUJ_ gender = Man: **1** - Woman: **2**
- Vision = Correct: **1** – Average but corrected: **2** – Average but uncorrected: **3**
- Vision_ assistance = None: **0** – Contact lens: **1** – Glasses: **2**
- Symptoms = Epileptic: **4** – Migraine: **3** – Recognized disorder attention deficit/Hyperactivity Disorder: **2** – Restless leg syndrome: **1** – None: **0** – Other **5**
- Level of study = Secondary school diploma **0**– High school diploma: **1** - 1 or 2 year undergrad, no diploma **2** – 2 years diploma: **3** – Bachelor degree: **4** – Master degree: **5** – PhD: **6**
- Level_ knowledge neuro = Level of knowledge about cognitive sciences/neuroscience, scale from **1** - I know nothing - to **5** - it is my specialty
- Meditation practice = No: **1** – Yes: **2**
- Laterality answered = Self reported laterality, Right: **1** – Left: **2** – Ambidextrous: **3**
- Manual activity = practice, Yes every day: **5** – Yes several times per week: **4** – Yes around one time per week: **3** – Yes less than one per week: **2** – Never: **1**
- Manual activity TXT = Text details Manual activity practice

Note that a problem in the recovery of raw data from this questionnaire for *Dataset B* resulted in the loss of the answer to the question “level of knowledge about cognitive sciences/neurosciences” for 13 participants.

#### Mental Rotation test

The Mental Rotation test was answered on paper at the beginning of the session. The test measures spatial visualisation abilities (SVA). In a study by Jeunet *et al*.^14^, MI-BCI control abilities were correlated to SVA [*r* = 0.696, *p* < 0.05]. Thus, we used the same french version^35^ of the mental rotation test by Vandenberg and Kuse^24^. The test is performed in two parts with a maximum time of 180 second each (in the “Performance.xlsx” file column time_ 1 and time_ 2) so 360 seconds in total and the score is computed on 40 points (column score).

#### Pre-experiment questionnaire

The pre-session questionnaire (the NeXT questionnaire in^25^) was filled-in on a computer at the beginning of the experiment. In this questionnaire we assigned a numerical code for the multiple choice questions. Each item corresponds to the name of a column in the “Performance.xlsx” file. It assesses the user’s states, notably:

- Pre-session states according to three factors (i.e. mood, mindfulness, and motivation) based on a subjective 5-point Likert scale in eight items (Appendix A in^25^).
  - PRE_ Mood
  - PRE_ Mindfulness
  - PRE_ Motivation
- A reported measure of sleep history and habits:
  - PRE_ Hours_ sleep_ last_ night = Number hours of sleep they had the night before
  - PRE_ Usual_ sleep = their usual amount of sleep.
- Emotional valence and arousal based on the Self-Assessment Manikin scale (SAM)^36^.
  - PRE_ Level_ of_ alertness = Participants’ self-rating of their level of alertness on a scale (from **1**: Sleepy to **5**: very excited).
- Stimulant consumption (stimulants, drug, meal…), namely:
  - PRE_ Stimulant_ doses_ 12 h = The amount of stimulant doses (coffee, tea, energy drink, coca-cola, etc.) they had during the last 12 hours;
  - PRE_ Stimulant_ doses_ 2 h = The doses they had during the last 2 hours;
  - PRE_ Stim_ normal = A rating comparing these doses with their usual consumption (with rather unusual, usually I take more: **1** - rather usual: **2** - rather unusual, usually I take less: **3**).
  - PRE_ Tabacco = The last time they used tobacco (today: **3** - yesterday evening: **2** - none: **1**).
  - PRE_ Tabacco_ normal = A rating comparing their usual consumption (with rather unusual, usually I take more: **1** - rather usual: **2** - rather unusual, usually I take less: **3**).
  - PRE_ Alcohol = The last time they drink alcohol (today: **3** - yesterday evening: **2** - none: **1**).
  - PRE_ Last_ meal = When they ate for the last time (less than 1 h ago: **1** - between 1 and 2 h ago: **2** - between 2 and 3 h ago: **3** - between 3 and 4 h ago: **4** - between 4 and 5 h ago: **5** - more than 5 h ago: **6**).
  - PRE_ Last_ pills = The last time they consumed drugs or medication (today: **3** - yesterday evening: **2** - none: **1**) and write what it was.
  - PRE_ Pills_ TXT = In text the description of what they take as drugs or medication.

For 13 participants from Dataset B, data for the questions (b) and (e) were lost due to a technical problem.

#### Post-experiment questionnaire

At the end of their session, participants were asked to complete a post-session state questionnaire retrieving five factors, such as mood, mindfulness, motivation, cognitive load, agentivity, based on subjective 5-point Likert scale in 21 items (Appendix A in^25^). The results of these questions are given in the columns of the same name, preceded by the word *POST_* in the “Performance.xlsx” file. Participants also rated on a scale whether the session fulfilled their expectations (column POST_ Expectations_ filled = from **1**: worse than I expected to **5**: better than I expected).

#### Index of Learning Style (ILS)

Participants completed the ILS at-home on a computer before the session. This questionnaire determines the participants’ preferred learning styles. It scores 4 scales (active/reflective, sensing/intuitive, visual/verbal, and sequential/global) based on 44 two-choice items^23^. The results of these questions are given in the 6 columns of the same name in the “Performance.xlsx” file.

#### The 5th edition of the 16 Personality Factors (16PF5)

The 16PF5 is an introspective psychometric questionnaire aiming to assess various dimensions of people’s personality profile with some cognitive traits^22^. This questionnaire contains 185 items and identifies 16 primary factors of personality, that are (A = Warmth, B = Reasonning, C = Emotional stability, E = Dominance, F = Liveliness, G = Rule-consciousness, H = Social boldness, I = Sensitivity, L = Vigilance, M = Abstractness, N = Privatness, O = Apprehension, Q1 = Openness to change, Q2 = Self reliance, Q3 = Perfectionism, Q4 = Tension and IM = Social desirability).

In addition to these 16 scales, the test highlights five broad factors (EX = Extroversion, AX = Anxiety, TM = Intransigence, IN = Independence, SC = Self control, and Interrogation). The whole test takes 35 to 50 minutes to complete, thus participants were asked to complete it online before coming. Note that due to technical issues, for the participant C83, results of the ILS and the 16Pf5 questionnaires were lost. The letter before each factor is the name of the columns in the “Performance.xlsx” file.

## Data Records

**Fig. 6:**
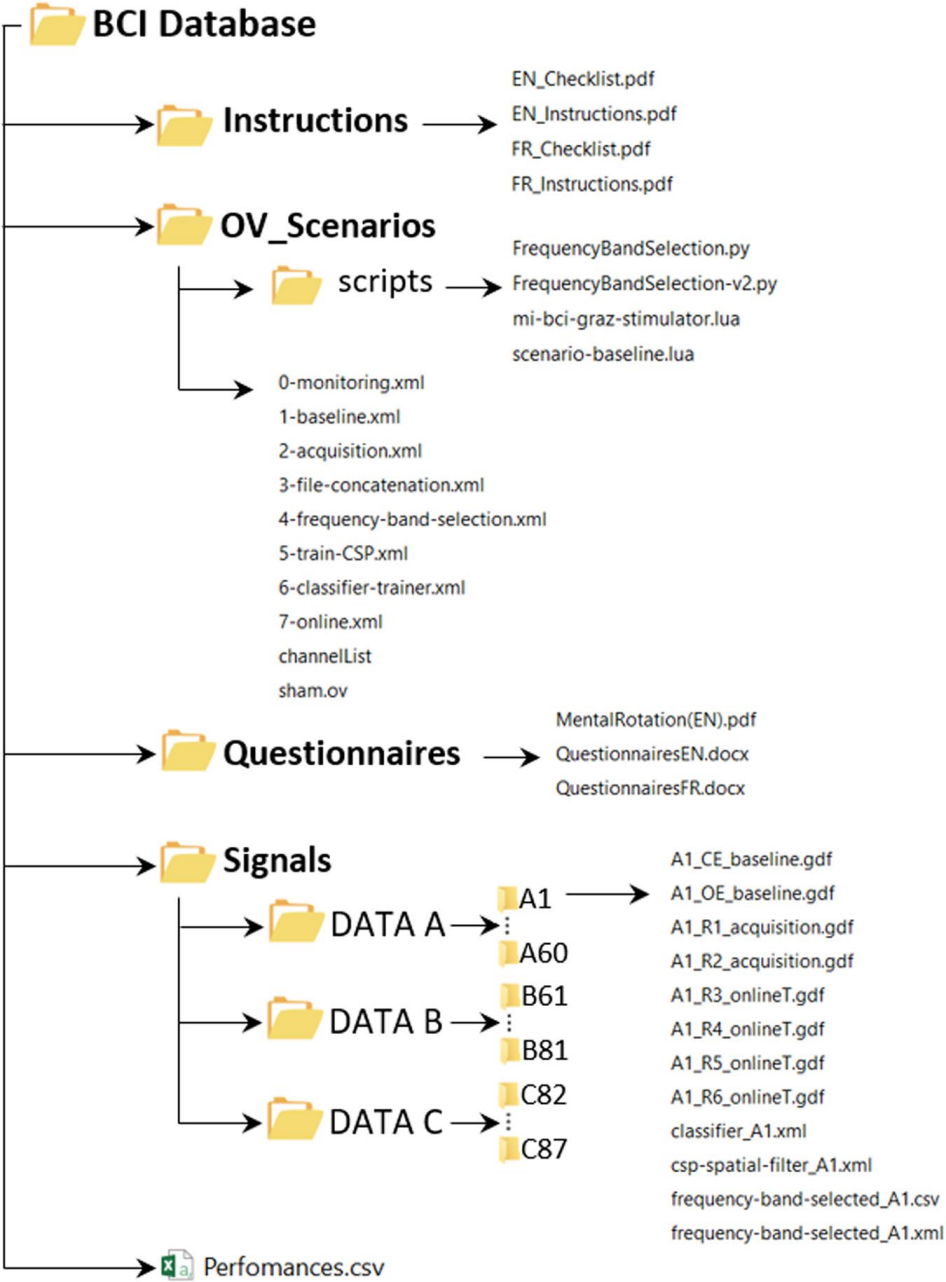
Final database structure, files, and naming.

A global compressed file (BCI Database.zip) containing the data of the two experiments is provided on the general-purpose open-access repository Zenodo at: 10.5281/zenodo.8089820^32^. It contains 4 folders named “Instructions”, “OV-scenarios”, “Questionnaires” and “Signals” and 1 file “Performances.xlsx”. A description of the different folders is provided in the following paragraphs, and Fig. 6 represents the database structure.

### Instructions

We provide the instructions read by experimenters during the experiments, in both English “EN-instruction.pdf” and French “FR-instructions.pdf”, as well as the checklist “En-Checklist.pdf” used during the experiments so that the experimenter would not forget anything during the experiment.

### OV-Scenarios

All the programs required to perform the online (real-time) BCI experiments (e.g., visual interfaces or EEG classification algorithms) were implemented using the free and open-source BCI OpenViBE platform^20^. On OpenViBE, the scenario is the name given to a set of OpenViBE modules used to perform a given step of a BCI protocol, e.g., acquire training data, calibrate a classifier or run an online BCI. We thus shared them all in the “OV-Scenarios” folder that contains the “OV-ScenariosA” and the “OV-ScenariosB” folders, for the scenarios used for Datasets A and B respectively. These folders contain the following 8 scenarios:

- Monitoring: *0-monitoring.xml* shows online signals acquired with the electrodes (see Fig. 2). Using this scenario, the experimenters checked the quality of the EEG signal and asked the participants to close their eyes to observe activity in the alpha band. To check the signal and for educational purpose, participants were also encouraged to clench their teeth and blink to observe the influence of artefacts on EEG data.
- Baseline: “*1-baseline.xml*” displays a fixation cross for a given duration (3 min in our experiment) while recording participants’ physiological data. It uses the *scenario-baseline.lua* script.
- Acquisition: “*2-acquisition.xml*” was used during runs R1 & R2. It displays the cues and sham feedback described in Figs. 4, 5 while recording the participants’ physiological data. The sham feedback is based on the *sham.ov* file.
- File concatenation: “*3-file-concatenation.xml*” concatenates the two files obtained from the two acquisition runs into one single file so that it can be processed by the two following scripts.
- Frequency band selection: “*4-frequency-band-selection.xml*” selects the most discriminant frequency band based on the concatenated file, with either the Blankertz’s algorithm^29^ - script *frequencyBandSelection.py* or the Benaroch’s variant with constraints^11^ - script *frequencyBandSelection-v2.py*. It saves the information obtained in a configuration file with extension “.cfg”.
- Train-CSP: “*5-train-CSP.xml*” uses the Common Spatial Pattern algorithm to optimize three pairs of spatial filters, using the concatenated file. It saves the information obtained in a configuration file with extension “.cfg”.
- Classifier-trainer: “*6-classifier-trainer.xml*” calibrates the Linear Discriminant Analysis classifier, with the concatenated file. It saves the information obtained in a configuration file with extension “.cfg”.
- Online: “*7-online.xml*” was used during runs R3 to R6. It displays the cues and feedback described in Figs. 4, 5 while recording the participants’ physiological data. It uses the *mi-bci-graz-simulator.lua* script.

The “OV-Scenarios” folder also contains the list of all electrodes used to record EEG signals, provided in the *“Channel-list.xml”* document, as well as the *“sham.ov”* file. It also contains the “scripts” folder containing the 2 Lua and the Python scripts associated to the OpenViBE scenarios described above.

### Questionnaires

We provide the Mental Rotation test used during the experiments in english “*MentalRotation(EN).pdf*”, as well as a translation of 4 questionnaires in “*TranslationQuestionnaires.docx*” notably the Demographic and Social information, the Pre and Post-session questionnaires, and the Index of Learning style. All of these questionnaires are also provided in French.

### Signals

The “Signals” folder contains 3 folders: *Dataset A* (60 participants), the *Dataset B* (21 participants) and the *Dataset C* (6 participants), and the “*Performance.xlsx*” file. Each Dataset contains a folder for each participant with 11 files in total. Among them, eight raw EEG recordings in *GDF* format (General Data Format for Biomedical Signals)^37^ with the open and closed eyes (OE/CE) EEG baseline, the first two acquisition runs and the four online training runs. Three files corresponding to the configuration files of the classifier output, the Common Spatial Pattern (CSP) spatial filter^31^ and the MDFB frequency bands selected are also provided in *.cfg* format.

### Performances

In the file “Performances.xlsx” the data of all participants are grouped together. The column SUJ_ ID is the anonymised number of the subject, The column SUJ_ gender is the gender of the subject and The column EXP_ gender is the gender of the experimenter who conducted the experiment. The column ‘comments’ refers to unexpected events or observations made by the experimenter (more details are given in the technical validation section). The online OpenViBE BCI classification performance obtained by each participant is given for each run in the column in Perf_ RUN_ 3, Perf_ RUN_ 4, Perf_ RUN_ 5 and Perf_ RUN_ 6. As mentioned in the section on BCI performance metrics and signal processing, the metric used to quantify BCI performance is the online TAcc. This is followed by the data from the 6 questionnaires. Each column is described in the method section.

### Missing Data

For participant A1, the two individual acquisition runs were missing, but the concatenated file that contains all the data about these two runs together was available so we extracted them. However they miss the end of trial trigger and the end of each run. For A59, the EEG data and filters for the last two runs (run R5 and R6) are missing, because the participant had to leave before the end of the experiment. He thus did not complete these two runs. For A11 and A9, only the first acquisition run was used to compute the frequency band selection algorithm, as the frequency band selection program could not find a discriminant band on the data of both runs. Both runs are nonetheless available and shared for each subject.

## Technical Validation

In this database, we have decided to share all the recorded trials and channels, including the noisy ones, together with the annotation that describes their quality. Infact, a branch of BCI and EEG research is dedicated to designing signal processing algorithms to detect, reject or clean noise in EEG signals^38^ or to designing machine learning algorithms robust to such noise. Noisy trials and channels could thus be used for this purpose, and would reflect typical signals recorded during real BCI use. To analyze our signals quality, we first performed an online signal quality analysis followed by an offline signal analysis.

### Online analysis

At the beginning of each BCI experiment, the EEG signal quality (e.g., raw signals, noise, electrode placement, artefacts occurrence) was carefully assessed by the trained experimenters. The experimenter notably visually checked each electrode signal for the absence of high frequency noise, or unusual signal patterns, and arranged the electrode montage and gel accordingly if needed. To ensure that EEG electrodes did measure a signal, the experimenters visually checked that blinks and jaw clenching did lead to visible artifacts in real-time EEG recordings. Then, to ensure that the EEG electrodes were measuring cortical signals, the experimenters also checked for the visual presence of the alpha rhythm amplitude increase in the occipital channels when the subjects closed their eyes. The quality of the EEG signals was also regularly checked visually by the experimenters between BCI runs, and the montage was adjusted if necessary. All unexpected events that occurred during runs (ex: R1, R3) or during Opened/Closed Eyes Baseline (BL OE/CE) are documented in the “comments” column in the “Performances” file. For example, there are the names of the electrodes whose signals were noisy based on the experimenter’s visual inspection. This also includes the possibility of artifacts in the run, such as noise heard outside the experimental room or the description of what the participants did (dozed off, moved, etc.). Such procedures would ensure a baseline quality for the recorded EEG signals. However, since EEG signals and sensors are very sensitive to noise and artifacts^39^, these procedures would not completely prevent the occurrence of noisy trials or channels at times.

### Offline analysis

#### BCI performance (TAcc)

**Fig. 7:**
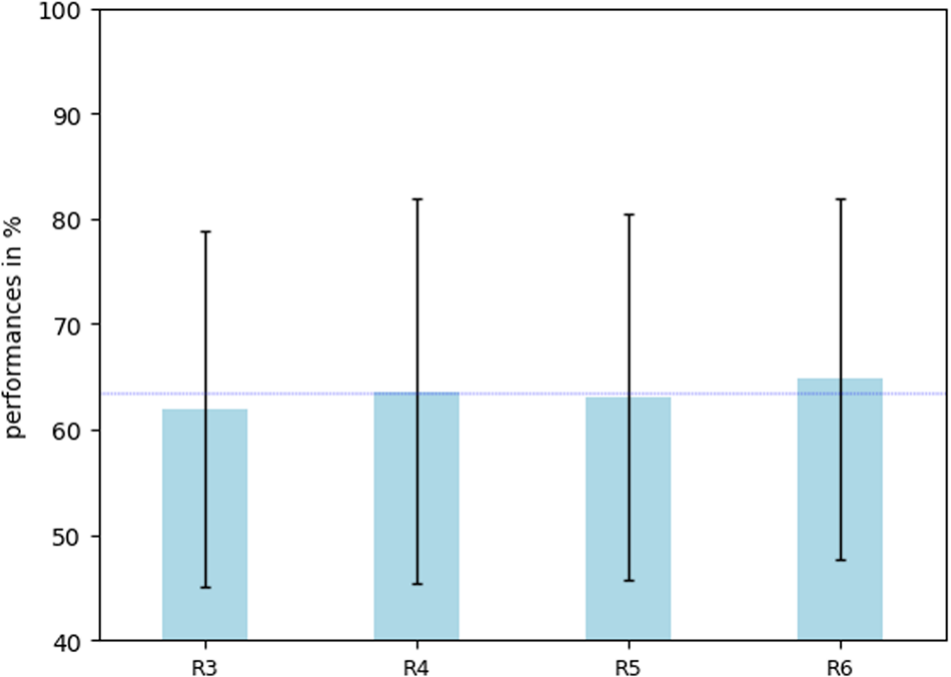
Histogram showing the average BCI performance of all participant per run. Blue line: mean = 63,37%.

**Fig. 8:**
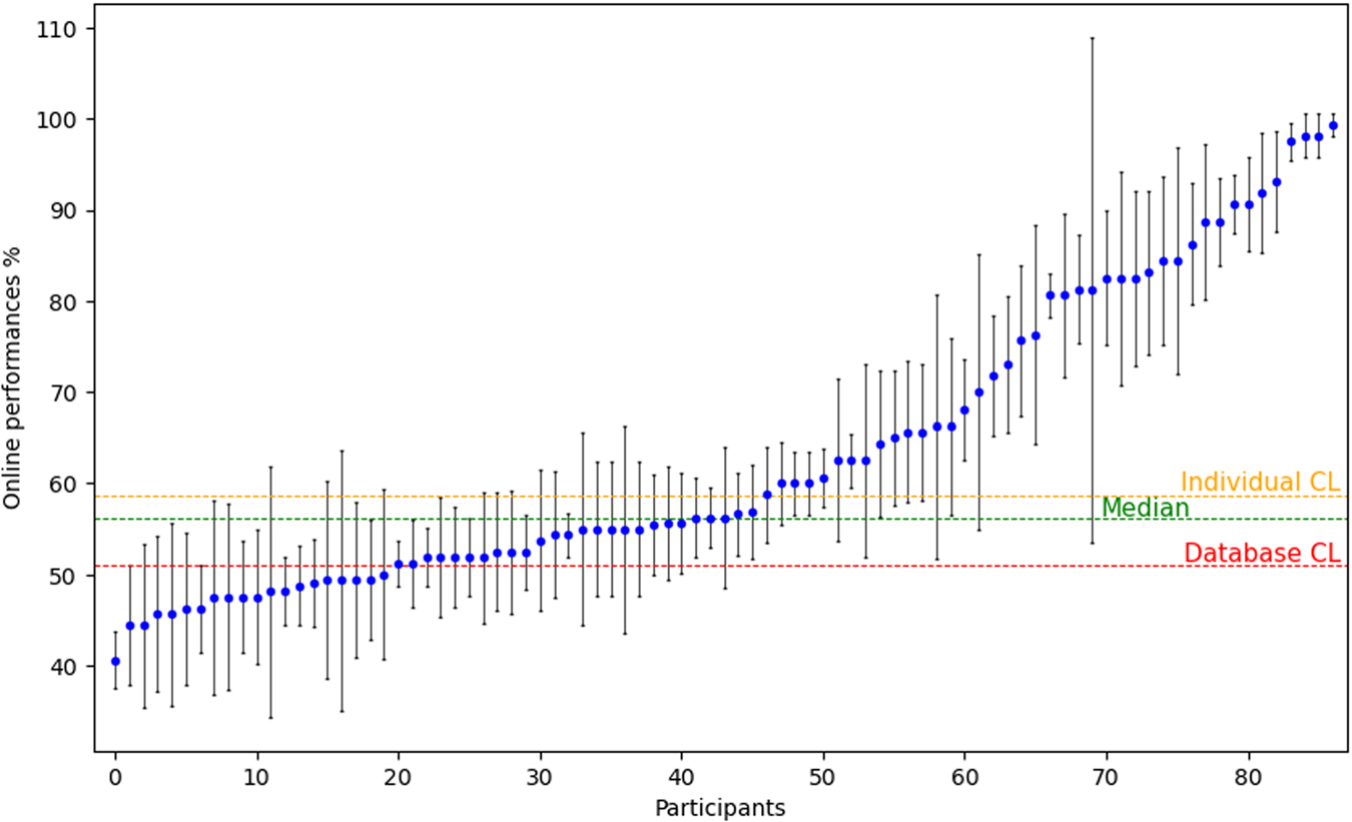
Scatter plot showing the average BCI performance of the 4 runs per subject. Blue points represent BCI performance averaged over the 4 recorded per participant. Connected black lines indicate the standard deviation per run. Individual chance level: 58,7%, Median: 57,5%, Database chance level: 51%^40^.

Figures 7, 8 report on the average performance obtained online by all participants, for each of them arranged in increasing TAcc in Fig. 8, and averaged for each run across participants in Fig. 7. Results show a relatively large proportion (55%) of participants with statistical chance level (CL) accuracy. This proportion is similar to the illiteracy rate reported in^16^ (53,7%). BCI performance varied largely between participants (Fig. 8) (mean 63.35% ± 17,36%) with (R1 = 61,92% ± 16,87%, R2 = 63,62% ± 18,24%, R3 = 63,14% ± 17,34%, R4 = 64,82% ± 17,16%). Performance ranged from 40% to almost perfect control 99,37%, individual chance level being 58,7% with p = 0.05^40^. For most participants, performance was also highly variable between runs. Specifically, the within-subject variability in performance between runs ranged from 1.25% to 27.7% (mean 7.13% ± 3.81%) see Fig. 8.

#### ERSP analysis

If our database is to be used for neurophysiological analyses, we would recommend rejecting noisy trials and channels. Using the tools available in OpenViBE and/or EEGLab^41^, the raw signals were all replayed and double-checked by an experimenter. Several problems were identified allowing a potential exclusion of certain subjects in future analyses, due to poor signal quality or equipment failure. We thus computed topographic event-related spectral perturbation (ERSP) between 8–30 Hz for each subject and phase, to check for possible unusual ERD/ERS patterns (i.e. abnormally low or high ERSPs compared to the average of all subjects). Specifically, abnormally high or low ERSP values 3 times higher than the mean were considered unusual. We computed the ERSP from EEGLab which is equivalent to the “band power method”^42^. With this method, the event-related spectral power at each time-frequency point is divided by the average spectral power in a 2 s pre-stimulus baseline period for each frequency band. The analysis of ERSP enabled us to visualize for which subjects there were major problems in the EEG signal. From that analysis, the following 7 participants were identified as potentially problematic for neurophysiological analyses:

- A04: technical problem concerning the FC5 electrode which generated large artifacts in the raw and filtered signals. ERSP analysis confirmed an unusual amplitude of power in the 8–30 Hz frequency band;
- A09: technical problem concerning the FC5 electrode which generated large artifacts in the raw and filtered signals. ERSP analysis confirmed an unusual amplitude of power in the 8–30 Hz frequency band;
- A17: technical problem related to the order in which the electrodes were recorded (as we used two amplifiers) in OpenViBE, resulting in both a problem with the arrangement of the electrodes and a disturbed EEG signal. ERSP analysis showed an unusual amplitude activity for all electrodes in 8–30 Hz;
- A29: possible disconnection or too high impedance of FC5 and CP5 electrodes. ERSP analysis showed unusual reduced amplitude of power for both electrodes in 8–30 Hz;
- A41: technical problem related to the order in which the electrodes were recorded (as we used two amplifiers) in OpenViBE, resulting in both a problem with the arrangement of the electrodes and a disturbed EEG signal (similar to A17). ERSP analysis showed an unusual amplitude activity for all electrodes in 8–30 Hz;
- B78: major artefacts occurred on the P4, C3, FC3 electrodes in different runs. ERSP analysis showed unusual increased amplitude of power for all these electrodes in 8–30 Hz;
- B79: dysfunction of C2 electrodes in the raw and filtered signal. ERSP analysis confirmed an unusual amplitude activity in the 8–30 Hz for the C2 electrode.

**Fig. 9:**
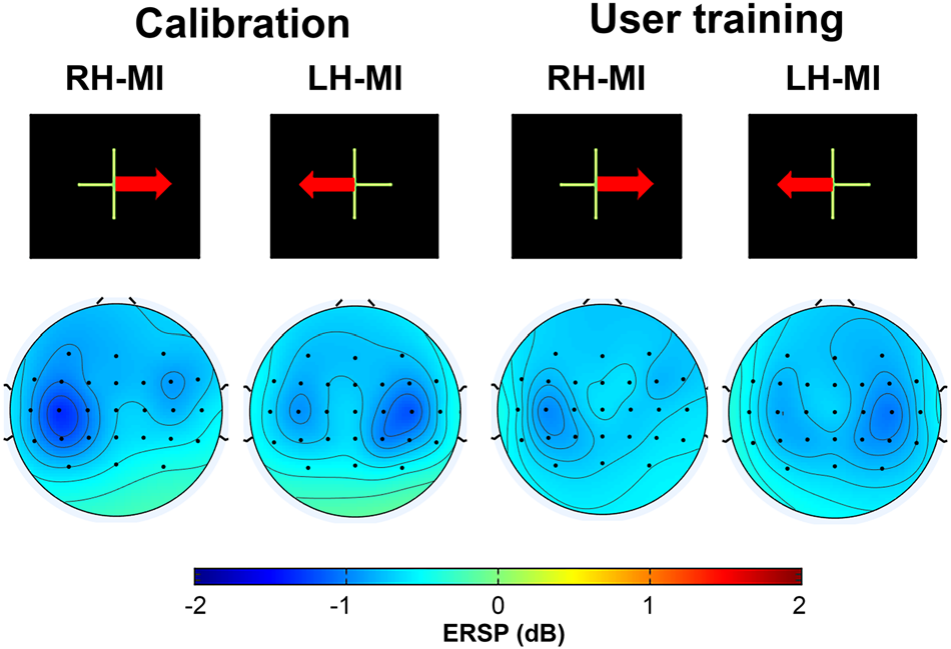
Topographic maps illustrating the ERD/ERS% (grand mean, n = 80) in the alpha/mu + beta band during both right and left motor imagery (MI) tasks in both the calibration and user training phases. The time window considered is from 3 to 8 seconds.

**Fig. 10:**
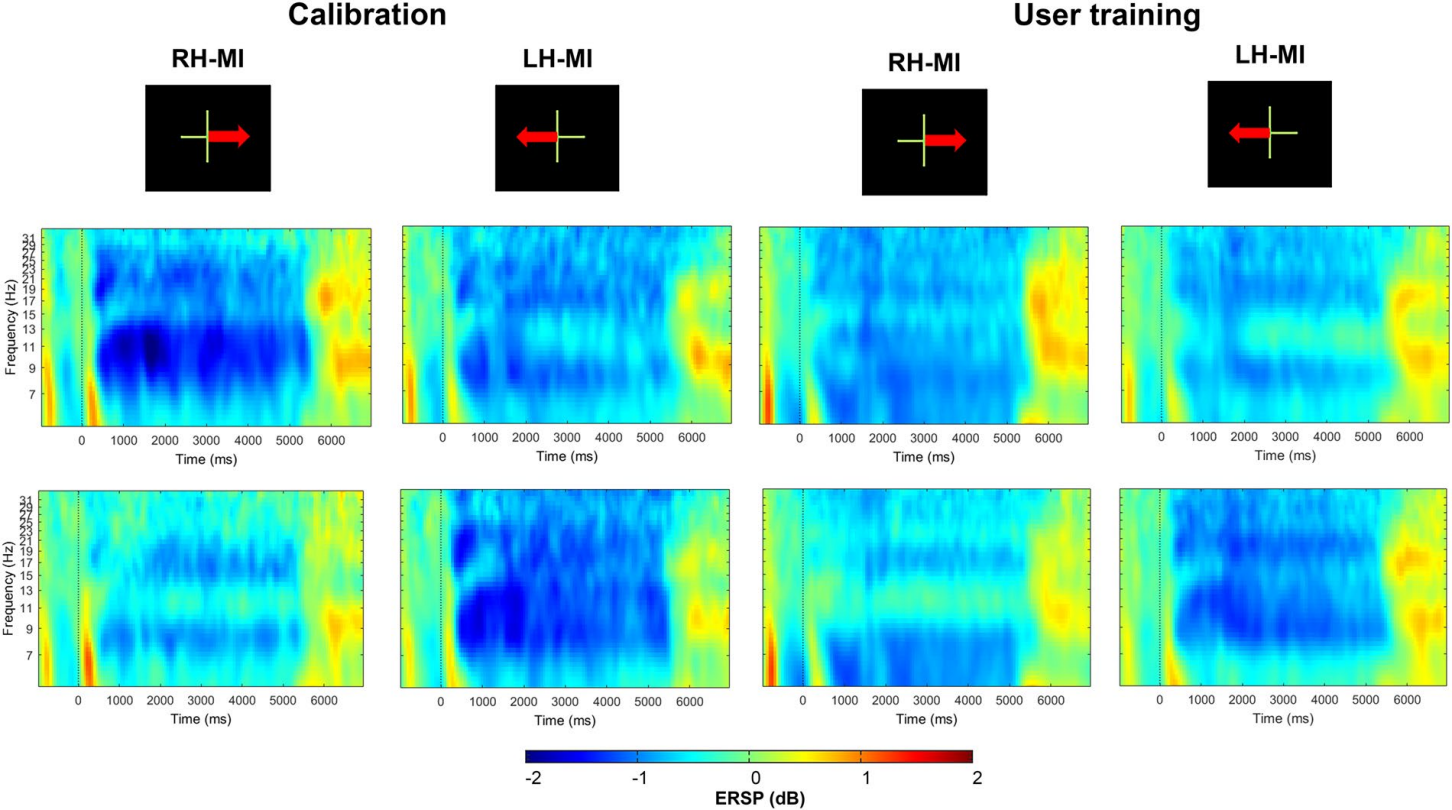
Time-course of the ERD and ERS. Strong ERD is represented by the blue, while strong ERS is depicted by red.

After having excluded the 7 subjects above with noisy data, we computed the average topographic maps and ERSP analysis for each class (left hand and right hand MI) over the remaining 80 subjects. The results show that there is an activation of the motor cortex with a controlateral ERD, i.e., covering either right and left-hand motor areas depending on the task, during the 5 s when the MI task was performed (see Figs. 9, 10). This result is in harmony with the literature^43,44^: the ERD phase is mainly observed during the MI task and represents an activation of the motor cortex. Note that a slightly bilateral desynchronization was observed for the left-hand MI.

Results in Figs. 9, 10 showed no significant difference between the calibration (first two runs) and the user training phase (last four runs) during the MI tasks, especially for contralateral electrodes (C3 and C4). This result is observed for both the right and left hand MI tasks.

Overall, our results show consistency with the literature, both in terms of classification performance for right-hand-left discrimination^12,43^ (Figs. 7, 8) and in terms of ERSP variation^43,44^ (Figs. 9, 10). In addition, a recent analysis by Rimbert *et al*.^45^, found that the left vs. rest and right vs. rest conditions showed good ERSPs for this database.

## Usage Notes

To analyse the raw EEG, we recommend using a standard open-source toolbox for EEG data, such as EEGLab^41^ (https://sccn.ucsd.edu/eeglab/index.php) in Matlab (The MathWorks Inc. Natick, MA, USA) or MNE Python^46^. EEG data also contain the triggers (a.k.a events) associated to each cue and phase of the BCI trials and runs. Such triggers are known as “stimulations” in OpenViBE. The OpenViBE stimulations we used in this database and their numeric values for the experiments are:

- ‘32769’ and ‘32770’ respectively indicate the beginning and the end of the run;
- ‘768’ indicates the start of the trial;
- ‘786’ indicates when the cross appears on screen;
- ‘33282’ indicates when the acoustic signal is provided;
- ‘769’ and ‘770’ represents the apparition of a cue respectively indicating Left-hand and Right-hand MI;
- ‘781’ indicates when the feedback period begins;
- ‘800’ indicates the end of the feedback period and end of the trial.

To know more about all OpenViBE stimulations, please refer to the website online (http://openvibe.inria.fr/stimulation-codes).

Note that there is a slight difference between the size of the acquisition and online files contained in the two datasets A and B. It is due to the different versions of OpenViBE used during the two experiments. *Dataset A* was collected with OpenViBE 2.1.0 and *Dataset B* with OpenViBE 2.2.0. For *Dataset C* Participants C83 and C85 were collected with OpenViBE 2.1.0 and the remaining 4 participants with OpenViBE 2.2.0.

## Data Availability

As described above, all the code of the experiments in available free and open-source. The OpenViBE scenarios are shared with the database^32^, while the free and open-source OpenViBE BCI platform itself can be downloaded there: (http://openvibe.inria.fr/). To access and download the free and open source OpenViBE BCI platform, go to: http://openvibe.inria.fr/. The source code for all versions of OpenViBE is available at http://openvibe.inria.fr/pub/src/, for each version you will find a README with build instructions in the archive you have downloaded. You can also find the sources at https://gitlab.inria.fr/openvibe/meta. To install the corresponding versions, which will allow you to reuse the scenarios of the experiments corresponding to this manuscript, you need to download older versions of OpenViBE. OpenViBE 2.1.0 (for dataset A) is available at http://openvibe.inria.fr/pub/bin/win32/ and OpenViBE 2.2.0 (for dataset B) is available at http://openvibe.inria.fr/pub/bin/win64/. You will then need to open all the scenarios provided in zenodo^32^ and you will be able to run them.
